# Supplementary material for: Microglia-mediated synaptic pruning in the nucleus accumbens during adolescence: A preliminary study of the proteomic consequences and putative female-specific pruning target
Source: bioRxiv. 2023 May 3:2023.05.02.539121. Preprint. [Version 1] doi: 10.1101/2023.05.02.539121 (PMC10187173; doi:10.1101/2023.05.02.539121)
Supplement: Supplement 1 [file NIHPP2023.05.02.539121v1-supplement-1.pdf]

bioRxiv preprint doi: https://doi.org/10.1101/2023.05.02.539121; this version posted May 3, 2023. The copyright holder for this preprint (which was not certified by peer review) is the author/funder, who has granted bioRxiv a license to display the preprint in perpetuity. It is made available under aCC-BY 4.0 International license.

Supp. Table 1

Gene	Sex	Log2FC
Slc4a1ap	F	0.41172
	M	-0.5752
Cyb5r3	F	-0.4866
	M	0.26419
Rpl27	F	0.33966
	M	-0.3207
Ube2z	F	0.77941
	M	-0.2103
Ncbp1	F	0.8934
	M	1.4857
Pccb	F	0.35973
	M	-0.3637

Supp. Table 1: DEPs regulated by NAc pruning shared between male and female rats
Only 1 of 7 DEPs regulated by NAc pruning, Ncbp1, was regulated in the same direction in male and female rats.
